# Association of triglyceride glucose index with incident diabetes among individuals with normal fasting triglycerides and fasting plasma glucose values: a general population-based retrospective cohort study

**DOI:** 10.3389/fendo.2025.1598171

**Published:** 2025-06-16

**Authors:** Yongchao Wang, Tiantian Cheng, Tie Zhang, Rubing Guo, Liang Ma, Wei Zhao

**Affiliations:** ^1^ Department of Clinical Laboratory, China-Japan Friendship Hospital, Beijing, China; ^2^ Department of Traditional Chinese Medicine Dermatology, Chaoyang District Taiyanggong Community Health Service Center, Beijing, China; ^3^ School of Public Health, Gansu University of Traditional Chinese Medicine, Lanzhou, China

**Keywords:** triglyceride glucose index, diabetes, cohort study, risk factors, inflection point

## Abstract

**Introduction:**

The triglyceride glucose (TyG) index has been proposed as a reliable surrogate marker for insulin resistance. Previous studies have demonstrated a significant positive association between the TyG index and diabetes risk. However, whether this association persists among individuals with normal fasting triglyceride (TG) and fasting plasma glucose (FPG) levels remains unclear. This study aims to evaluate the relationship between the TyG index and diabetes in this specific population utilizing a large-scale population-based dataset.

**Methods:**

A total of 155,337 subjects (74,622 males and 80,715 females) were included in this retrospective cohort study. The TyG index was calculated using the formula: ln [TG (mg/dL) × GLU (mg/dL)/2]. Cox regression analyses were utilized to estimate hazard ratios (HR) and 95% confidence intervals (CI). A generalized additive model (GAM), combined with smooth curve fitting, was employed to investigate potential nonlinear associations. Subgroup analyses were stratified by age, sex, body mass index, and family history of diabetes.

**Results:**

During a 3.13-year mean follow-up, 1127 subjects (0.73%) developed diabetes. After adjusting for confounding variables, subjects in the highest quartile of baseline TyG index exhibited an increased risk of diabetes compared to those in the lowest quartile (adjusted HR: 3.80; 95% CI: 2.41-5.99; P for trend < 0.001). Furthermore, a nonlinear threshold association was identified, with an inflection point at a TyG index value of 8.41. For subjects with a TyG index greater than 8.41, the adjusted HR was 15.33 (95% CI: 7.3-32.2, P < 0.001). Subgroup analysis revealed a stronger positive correlation in individuals aged <60 years.

**Discussion:**

This large-scale study demonstrates a robust independent association between the TyG index and diabetes risk in individuals with normal FPG and TG levels. Our findings suggest that a TyG index exceeding 8.41 correlates with a progressively higher risk of diabetes development. These results collectively suggest the TyG index could serve as a clinically relevant predictor of diabetes incidence in this population.

## 1 Introduction

Diabetes, a global epidemic, is driven by dysregulated blood glucose metabolism (1–3). It is influenced by genetics, diet, lifestyle and environmental factors (4). Its global prevalence has risen sharply in recent decades. According to the International Diabetes Federation, approximately 537 million people had diabetes in 2021, with projections indicating this number could surge to 783 million by 2045, imposing immense strain on healthcare systems worldwide (5). Insulin resistance (IR), a key marker of metabolic syndrome and a key risk factor for diabetes, disrupts glucose metabolism in muscle, adipose, and liver (6). While the hyperinsulinemic-euglycemic clamp (HIEC) remains the gold-standard method for assessing IR, its high cost limits widespread clinical application (7–10).

The triglyceride glucose (TyG) index, a biomarker derived from fasting triglyceride (TG) and fasting plasma glucose (FPG) levels, is strongly correlated with insulin resistance (IR) and has been extensively validated as a relevant surrogate marker for IR (11, 12). Growing evidence links the TyG index to the pathogenesis of cardiovascular and metabolic disorders, including hypertension, myocardial infarction, and ischemic stroke (13, 14). Additionally, multiple studies further identified a significant positive correlation between diabetes and the TyG index (14–21). However, despite the fact that a substantial subset of individuals with normal TG and FPG levels progresses to diabetes without overt metabolic abnormalities, the predictive role of the TyG index in this specific population remains underexplored (22). This retrospective cohort study aims to assess the relationship between the TyG index and the prevalence of diabetes in individuals with baseline-normal TG and FPG levels utilizing a large-scale dataset.

## 2 Methods

### 2.1 Data source and study population

The data utilized in this *post hoc* analysis were extracted from a computerized database (www.datadryad.org) established by the Rich Healthcare Group in China, with an overview of the design provided elsewhere (23) (Dryad, https://doi.org/10.5061/dryad.ft8750v). This comprehensive database includes medical records for adult aged ≥20 years from 11 cities in China who underwent health examinations between 2011 and 2016. In total, the database encompasses 685,277 participants who had at least two visits.

**Figure 1:**
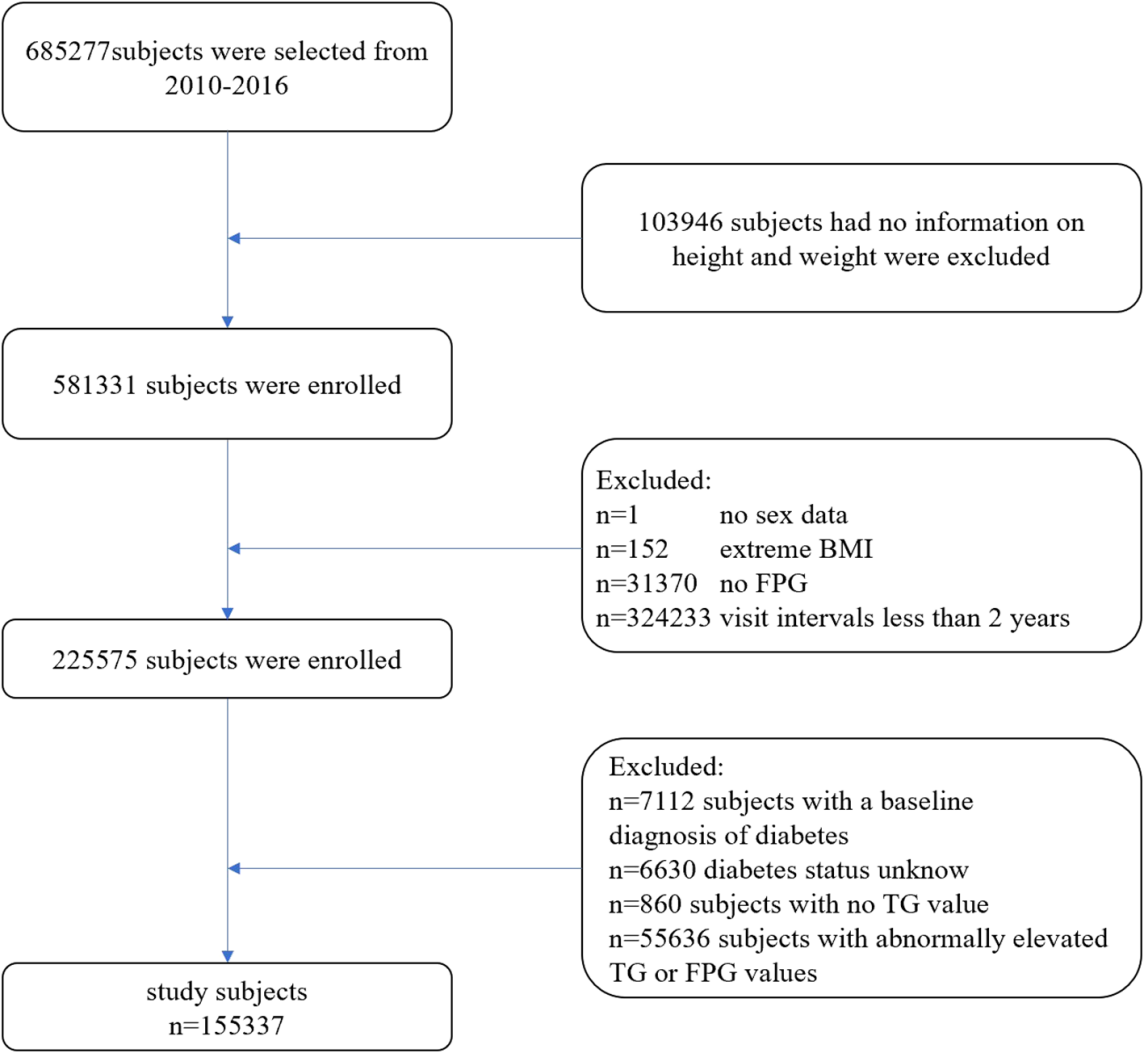
Flow diagram of subjects included in the cohort study.

The exclusion criteria applied in this study were as follows (**Figure 1**): (1) Participants without available information on weight and height; (2) Participants lacking data on sex, FPG values, TG values, or exhibiting extreme body mass index (BMI) values (<15 kg/m² or >55 kg/m²); (3) Participants with abnormally elevated FPG values (≥6.1 mmol/L) or TG values (≥1.7 mmol/L); (4) Participants with a follow-up interval of less than 2 years; (5) Participants with a baseline diagnosis of diabetes; and (6) Participants with unknown diabetes status during follow-up. Ultimately, 155,337 participants (74,622 males and 80,715 females) were included in this study.

### 2.2 Data collection

All participants were requested to complete a detailed questionnaire that included demographic characteristics such as age, sex, and family history of diabetes. Professional operators recorded the participants’ height, weight, and blood pressure at the health check center. Participants’ weight was measured in light clothing without shoes to the nearest 0.1 kg, while height was measured to the nearest 0.1 cm. Blood pressure was assessed using standard mercury sphygmomanometers. Professional staff collected fasting venous blood samples from participants after a minimum fasting period of 10 hours. Laboratory tests, including total cholesterol (TC), TG, FPG, high-density lipoprotein cholesterol (HDL-C), low-density lipoprotein cholesterol (LDL-C), alanine aminotransferase (ALT), and aspartate aminotransferase (AST), were performed using an automated analyzer (Beckman, 5800). FPG levels were measured by the glucose oxidase method using the same automated analyzer.

### 2.3 Definition

Body mass index (BMI) was calculated using the formula: weight (kg)/height (m)^2. The TyG index was calculated using the formula: ln [fasting plasma glucose (mg/dL) × fasting triglycerides (mg/dL)/2]. The TyG-10 index was calculated as the TyG index multiplied by 10. Diabetes was diagnosed based on a fasting plasma glucose level of ≥7.00 mmol/L and/or self-reported diabetes during the follow-up period.

### 2.4 Statistical analysis

Data were analyzed using the R software package (http://www.r-project.org, version 4.2.0, R Foundation) and EmpowerStats (www.empowerstats.net, X&Y Solutions, Inc., Boston, Massachusetts). A p-value of < 0.05 (two-sided) was considered statistically significant.

In this research, baseline indicators were grouped according to the quartiles of the TyG index. Categorical data are presented as numbers (percentages). Continuous variables with a normal distribution are reported as mean ± standard deviation (SD), while skewed continuous variables are presented as medians (interquartile range [IQR]). To assess the presence of statistically significant differences among the quartiles, the chi-square test was employed for categorical variables, the Kruskal-Wallis H test was utilized for skewed variables, and one-way analysis of variance (ANOVA) was applied for variables with a normal distribution (**Table 1**).

**Table 1:** Baseline Characteristics of participants (N =155337).

TyG index quartile	Q1(4.6-7.8) (n=38833)	Q2(7.8-8.1) (n=38786)	Q3(8.1-8.4) (n=38882)	Q4(8.4-9) (n=38836)	P value
Age (years)	37.0 ± 9.7	39.3 ± 11.3	41.7 ± 12.6	44.7 ± 13.4	<0.001
Gender, n (%)					<0.001
male	11936 (30.7)	16715 (43.1)	21139 (54.4)	24832 (63.9)	
female	26897 (69.3)	22071 (56.9)	17743 (45.6)	14004 (36.1)	
BMI (kg/m2)	21.2 ± 2.5	22.0 ± 2.9	22.9 ± 3.0	24.1 ± 3.2	<0.001
SBP (mmHg)	112.0 ± 13.9	115.1 ± 14.9	118.2 ± 15.6	122.1 ± 16.3	<0.001
DBP (mmHg)	69.8 ± 9.5	71.6 ± 9.9	73.5 ± 10.3	75.8 ± 10.6	<0.001
FPG (mmol/L)	4.6 ± 0.5	4.8 ± 0.5	4.9 ± 0.5	5.1 ± 0.5	<0.001
TG (mmol/L)	0.5 ± 0.1	0.8 ± 0.1	1.0 ± 0.1	1.4 ± 0.2	<0.001
HDL-C (mmol/)	1.5 ± 0.3	1.5 ± 0.3	1.4 ± 0.3	1.3 ± 0.3	<0.001
ALT (U/L)	13.5 (10.7-18.2)	15.0 (11.4-21.2)	17.0 (12.8-25.0)	20.2 (14.7-29.8)	<0.001
LDL-C (mmol/L)	2.4 ± 0.5	2.6 ± 0.6	2.8 ± 0.6	2.9 ± 0.7	<0.001
AST (U/)	20.0 (17.0-23.1)	20.5 (17.8-24.2)	21.4 (18.1-25.6)	23.0 (19.1-27.1)	<0.001
BUN (mmol/L)	4.5 ± 1.2	4.6 ± 1.2	4.6 ± 1.2	4.7 ± 1.2	<0.001
TC (mmol/L)	4.2 ± 0.7	4.5 ± 0.8	4.7 ± 0.8	4.9 ± 0.9	<0.001
SCR (umol/L)	64.1 ± 13.8	67.3 ± 16.3	69.9 ± 15.2	72.3 ± 16.0	<0.001
year of follow-up (year)	3.2 ± 1.0	3.2 ± 1.0	3.1 ± 0.9	3.0 ± 0.9	<0.001
TyG index	7.6 ± 0.2	8.0 ± 0.1	8.3 ± 0.1	8.6 ± 0.1	<0.001
Censor of diabetes at follow-up, n (%)					<0.001
No	38748 (99.8)	38634 (99.6)	38633 (99.4)	38195 (98.3)	
Yes	85 (0.2)	152 (0.4)	249 (0.6)	641 (1.7)	
Family history of diabetes, n (%)					0.014
No	38132 (98.2)	38017 (98.0)	38066 (97.9)	38030 (97.9)	
Yes	701 (1.8)	769 (2.0)	816 (2.1)	806 (2.1)	

Continuous variables with a normal distribution were reported as mean ± standard deviation (SD), whereas skewed continuous variables were presented as medians (interquartile range [IQR]). Categorical data are presented as n (%). To assess the presence of statistically significant differences among the quartiles, the chi-square test was employed for categorical variables, the Kruskal-Wallis H test was used for skewed variables, and one-way analysis of variance (ANOVA) was applied for variables with a normal distribution.

TyG index, the triglyceride glucose index; BMI, body mass index; SBP, systolic blood pressure; DBP, diastolic blood pressure; FPG, fasting plasma glucose; TG, triglyceride; TC, total cholesterol; LDL-C, low-density lipid cholesterol; HDL-C, high-density lipid cholesterol; BUN, blood urea nitrogen; SCR, serum creatinine; ALT, alanine aminotransferase; AST, aspartate aminotransferase.

In this study, we employed multivariate Cox regression to evaluate the relationship between the TyG index and the prevalence of diabetes, yielding hazard ratios (HRs) for diabetes with the corresponding 95% confidence interval (CI) after controlling for confounding variables. Three adjusted models were utilized for the multivariate Cox regression analysis: Model I was adjusted for age and sex; Model II was adjusted for age, sex, BMI, systolic blood pressure (SBP), and diastolic blood pressure (DBP); and Model III was adjusted for age, sex, BMI, SBP, DBP, HDL-C, ALT, AST, BUN, serum creatinine (SCR), and family history of diabetes. All unadjusted and adjusted multivariable models are presented in **Table 2**. A generalized additive model (GAM) incorporating a smoothing function was employed to identify potential nonlinear associations between the TyG index and diabetes. Additionally, a threshold effect analysis of the TyG index in relation to diabetes was conducted using piecewise linear regression to explore the inflection point of the TyG index. A hierarchical Cox regression model was utilized for subgroup analysis, and the likelihood ratio test was employed to assess interactions between the subgroups.

**Table 2:** Relationship between TyG index and diabetes.

Outcome	Crude model		Model I		Model II		Model III	
	HR (95% CI)	P-value	HR (95% CI)	P-value	HR (95% CI)	P-value	HR (95% CI)	P-value
TyG index	11.81 (9.86, 14.15)	<0.001	6.49 (5.39, 7.82)	<0.001	4.14 (3.42, 5.01)	<0.001	5.21 (3.59, 7.54)	<0.001
TyG-10 index	1.28 (1.26, 1.30)	<0.001	1.21 (1.18, 1.23)	<0.001	1.15 (1.13, 1.17)	<0.001	1.15 (1.11, 1.19)	<0.001
TyG index quartile								
Q1	Reference		Reference		Reference		Reference	
Q2	1.99 (1.52, 2.59)	<0.001	1.57 (1.20, 2.04)	0.001	1.33 (1.01, 1.73)	0.039	1.52 (0.92, 2.50)	0.100
Q3	3.47 (2.71, 4.44))	<0.001	2.22 (1.73, 2.84)	<0.001	1.63 (1.27, 2.10)	<0.001	1.49 (0.92, 2.43)	0.108
Q4	9.75 (7.78, 12.23)	<0.001	5.15 (4.08, 6.49)	<0.001	3.23 (2.55, 4.09)	<0.001	3.80 (2.41, 5.99)	<0.001
P for trend	<0.001		<0.001		< 0.001		<0.001	

Model I was adjusted for age and gender.

Model II was adjusted for age, gender, BMI, SBP and DBP.

Model III was adjusted for age, gender, BMI, SBP, DBP, HDL-C, ALT, AST, BUN, SCR and family history of diabetes.

CI, confidence interval; HR, hazard ratios; TyG-10 index, TyG index × 10;other abbreviations as in **Table 1**.

## 3 Results

### 3.1 Baseline characteristics of individuals

The original cohort comprised 685,277 participants. After applying exclusion criteria, 529,940 individuals were excluded. Ultimately, 155,337 participants (74,622 males and 80,715 females) with normal levels of FPG and TG were enrolled in this retrospective cohort study. The mean (SD) age of the participants included in this study was 40.7 (12.2) years, with a mean TyG index of 8.12 (0.42). The baseline characteristics stratified by TyG index quartiles are presented in **Table 1**. Participants in higher TyG index quartiles tended to be older, male, and exhibit higher body mass index (BMI), blood pressure, and elevated levels of FPG, TG, TC, ALT, AST, SCR, and LDL-C. All of the aforementioned differences were statistically significant (P < 0.001).

### 3.2 Incidence of diabetes

During a mean follow-up of 3.13 years, 1,127 participants (0.73%) developed diabetes, with 85 (0.2%) in group Q1, 152 (0.4%) in group Q2, 249 (0.6%) in group Q3, and 641 (1.7%) in group Q4. A significant increase in diabetes risk was observed with ascending TyG index quartiles (P < 0.001) (**Table 1**).

### 3.3 Association of normal triglyceride levels with diabetes mellitus type 2 risk

Multivariate Cox regression models were established to evaluate the association between the TyG index and diabetes risk (**Table 2**). Three progressively adjusted models were constructed, with covariates detailed in **Table 2**. In the crude model, the TyG index exhibited a strong positive correlation over time with the risk of diabetes [HR: 11.81 (95% CI: 9.86, 14.15), P<0.001]. This association remained significant even after adjusting for potential confounding variables. The adjusted HR (95% CI) for model 3 was 5.21 (3.59, 7.54) (P<0.001), indicating that a 421% increase in diabetes risk corresponds to a one-unit increase in the baseline TyG index. To better assess the elevated risk of diabetes associated with minor increases in the TyG index, we created the TyG-10 index using the same analysis. The corresponding HR (95% CI) for model 3 was 1.15 (1.11, 1.19), suggesting that the risk of diabetes increased by 15% for each 0.1 unit increase in the baseline TyG index, after accounting for all significant confounders. Additionally, multivariate Cox regression analysis was conducted treating the TyG index as a categorical variable. In the adjusted model 3, the HR and 95% CI for the highest quartile of the TyG index was 3.80 [(2.41, 5.99), P<0.001] compared to the lowest quartile (P for trend <0.001).

### 3.4 Threshold effect analysis of TyG index on incident diabetes

**Figure 2:**
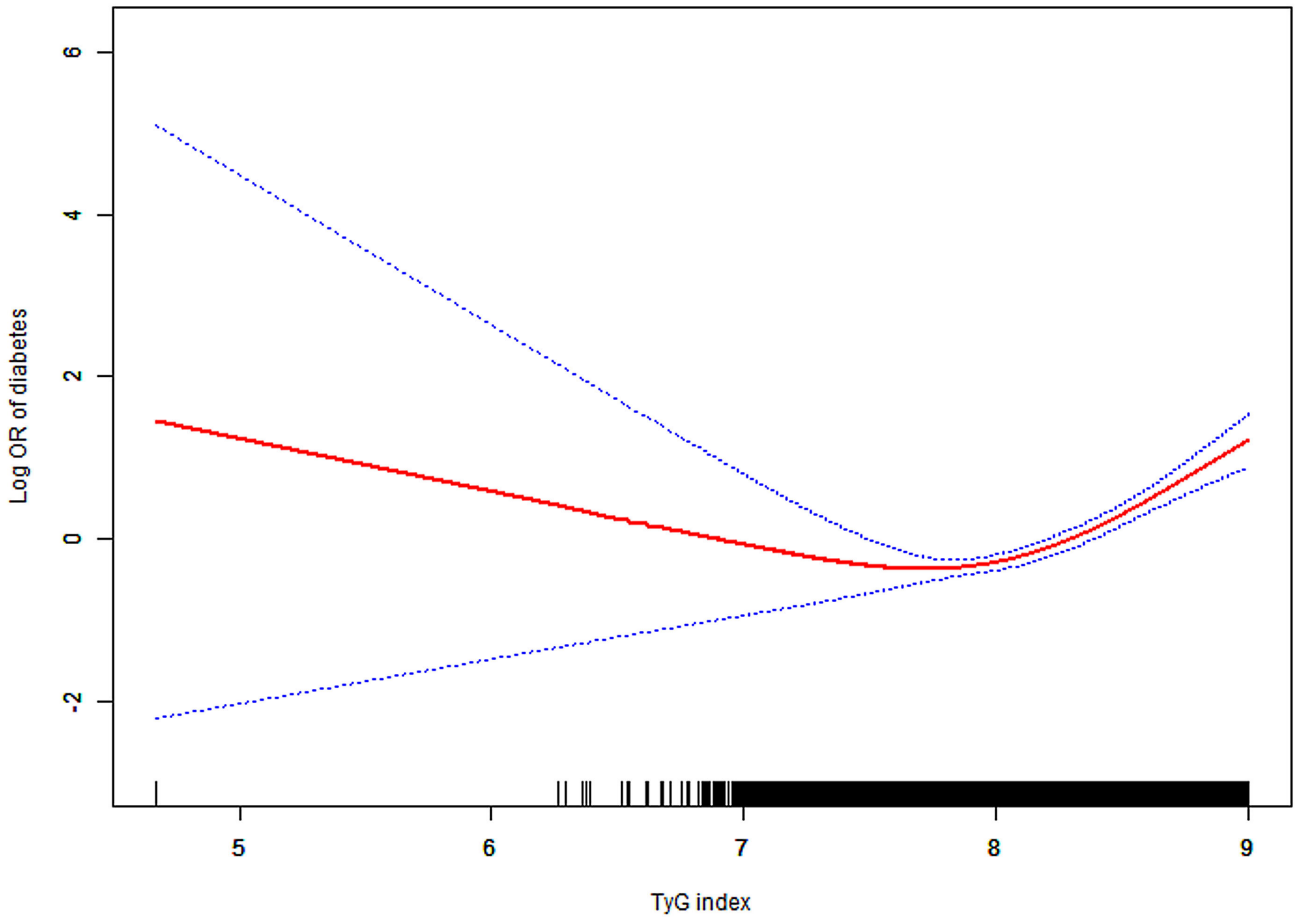
Association between TyG index and diabetes. A threshold, nonlinear association between TyG index and diabetes was found in a generalized additive model (GAM). Solid red line represents the smooth curve fit between variables. Blue bands represent the 95% of confidence interval from the fit. All adjusted for age, gender, BMI, SBP, DBP, HDL-C, ALT, AST, BUN, SCR and family history of diabetes. Abbreviations: as in **Table 1**.

After adjusting for age, gender, BMI, SBP, DBP, HDL-C, ALT, AST, BUN, SCR, and family history of diabetes, a threshold nonlinear association between the TyG index and diabetes was identified using a generalized additive model (GAM) (**Figure 2**).

We employed Piece-wise Linear Regression to evaluate the nonlinear threshold relationship between the TyG index and diabetes risk (**Table 3**). This analysis revealed an inflection point at the TyG index of 8.41. For TyG index values ≥8.41, the adjusted HR was 15.33 (95% CI: 7.30–32.20; P < 0.001). Conversely, for TyG index values <8.41, no significant association was observed (adjusted HR: 1.64; 95% CI: 0.95–2.83; P = 0.075).

**Table 3:** Threshold effect analysis of tyg index and diabetes using piece-wise linear regression.

	TyG index		TyG-10 index	
Inflection point	<8.41	≥8.41	<84.1	≥84.1
HR	1.64	15.33	1.05	1.31
95%CI	0.95 to 2.83	7.30 to 32.20	0.99 to 1.11	1.22 to 1.41
P-value	0.075	<0.001	0.083	<0.001

Adjust for age, gender, BMI, SBP, DBP, HDL-C, ALT, AST, BUN, SCR and family history of diabetes.

CI, confidence interval; HR, hazard ratios; TyG-10 index, TyG index × 10; other abbreviations as in **Table 1**.

### 3.5 Subgroup analyses

**Figure 3:**
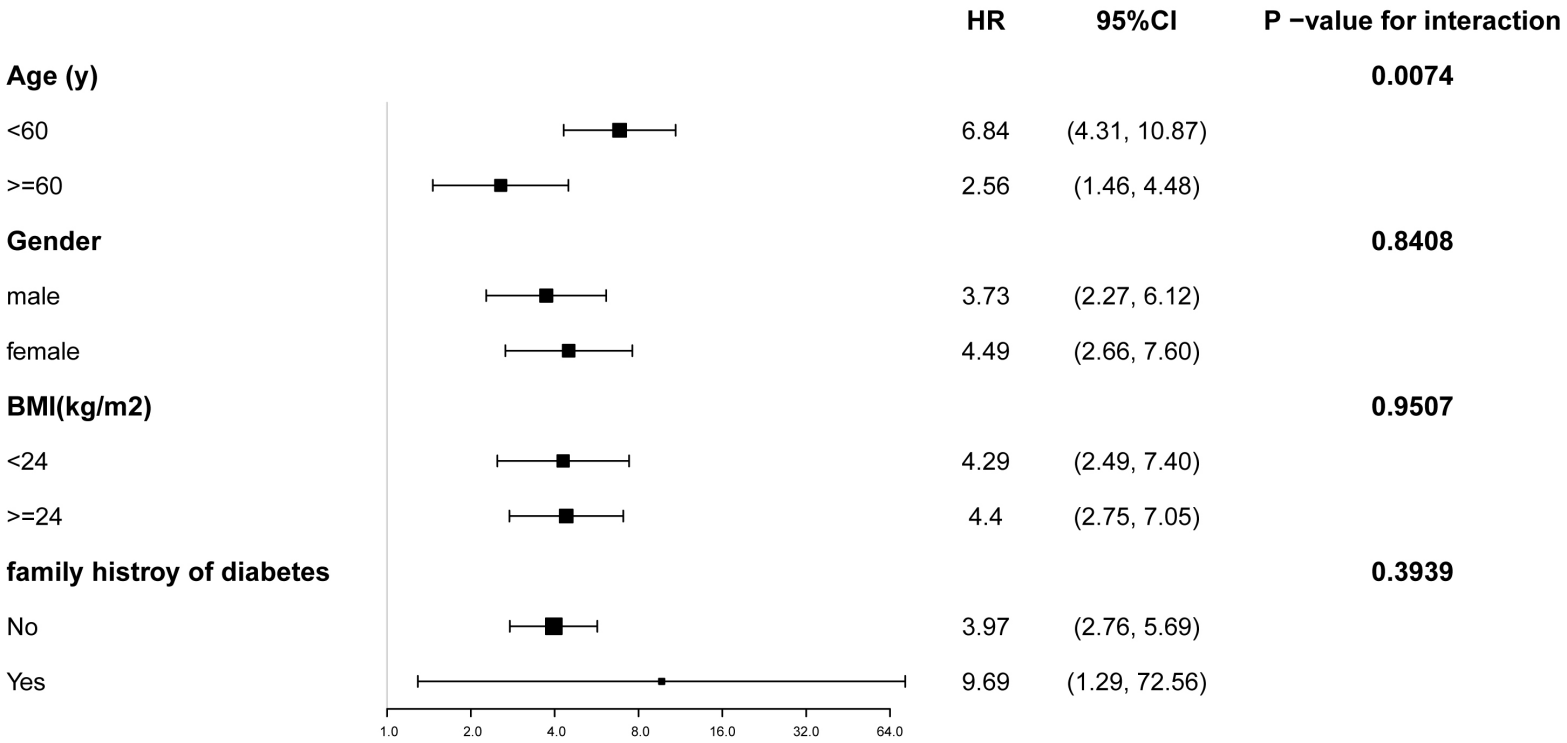
Subgroup analysis of the relationship between TyG index and diabetes. Adjustments were made based on age, gender, BMI, SBP, DBP, HDL-C, ALT, AST, BUN, SCR and family history of diabetes, except the subgroup variable. CI, confidence interval; HR, hazard ratios; other abbreviations as in **Table 1**.

Participants were stratified by age, sex, BMI, and family history of diabetes. **Figure 3** illustrates the TyG index’s association with diabetes risk across prespecified and exploratory subgroups. Adjusted analyses (Model 3) revealed a significant positive relationship between elevated TyG index and diabetes incidence in all subgroups (*P* < 0.05). Notably, a significant interaction was observed for the age subgroup (P for interaction = 0.0074) with markedly higher diabetes risk in participants aged <60 years (HR: 6.84; 95% CI: 4.31–10.87) compared to those aged ≥60 years (HR: 2.56; 95% CI: 1.46–4.48).

## 4 Discussion

In this large population-based retrospective cohort study, we observed an independent positive association between the TyG index and diabetes incidence among participants with normal FBG and TG levels, even after full adjustment for confounders. And each 0.1-unit increase in the TyG index correlated with a 15% higher risk of diabetes. We further revealed a significant interaction between the TyG index and age concerning the risk of diabetes. Notably, a nonlinear curve with an inflection point at 8.41 was identified between the TyG index and the incidence of diabetes.

Diabetes, a severe metabolic disorder, imposes significant physical and psychological burdens on patients. Unfortunately, a study conducted by Ogurtsova K et al. revealed that nearly half of adults with diabetes are unaware of their condition (24). Insulin resistance (IR), an important risk factor for diabetes, is defined as a disorder of glucose homeostasis (25–27). Although the hyperinsulinemic-euglycemic clamp (HIEC) method is the gold standard for evaluating IR, its clinical utility is limited by cost and procedural complexity (28, 29). While the TyG index, which is a simple and inexpensive surrogate marker of IR, is well correlated with the HIEC-measured IR (30, 31).

Multiple studies have reported a significant positive correlation between the TyG index and diabetes across diverse populations (14–21, 32, 33). A 12-year longitudinal cohort study involving 4,285 participants reported that the adjusted HR for the highest quartile of the TyG index was 3.59 (95% CI: 2.65-4.88) compared to the lowest quartile (15). Similarly, a cross-sectional study of 661 pregnant women in the United States indicated a positive association between the TyG index and gestational diabetes mellitus (GDM) after adjusting for all confounding variables, with an odds ratio (OR) of 3.43 (95% CI: 1.20-9.85, P = 0.0216) (16). Additionally, a 20-year retrospective study involving 862 elderly Chinese men revealed an independent correlation between an elevated TyG index and an increased risk of incident diabetes, with a multivariable HR of 1.525 (95% CI: 1.290-1.804) (17). A large longitudinal study of 15,012 prediabetic adults, conducted by Bo Chen et al., further confirmed this association with an adjusted HR of 1.86 (95% CI: 1.7–2.04) (19). Notably, in another cross-sectional study of 10,471 subjects, Bokun Kim et al. observed that the risk of diabetes increased with a rising TyG index. The TyG index cut-off values for metabolically obese, normal weight (MONW) individuals had odds ratios of 2.370 (2.021–2.778, p < 0.001) for males and 2.249 (1.937–2.610, p < 0.001) for females, compared to metabolically healthy, normal weight (MHNW) and pre-MONW individuals in the fully adjusted model (20). Furthermore, a comprehensive evaluation of meta-analyses conducted by Sandeep Samethadka Nayak et al. identified a significant relationship between the TyG index and type 2 diabetes mellitus (T2DM), although significant heterogeneity was observed (21).

The aforementioned studies have not specifically addressed subjects with normal FPG and TG levels, despite the clinical relevance of TyG index–diabetes associations in this population. Our findings align with prior research while uniquely demonstrating the association persists even among those with normal FPG and TG values. Notably, a 2021 cohort study published in Endocrine reported a TyG index threshold of 8.51 for the risk of T2DM (34).While a prospective cohort study from Korea including 4820 subjects without T2DM, revealed that the patients with a TyG index value ≥ 8.31 had an elevated risk of developing T2DM (32). Similarly, a large white European population-based prospective cohort study also found an increasing risk of T2DM in participants with a TyG index of 8.31 (33). These closely align with our identified inflection point of 8.41.

A cohort study involving 5,575 subjects (4,422 males and 1,153 females) conducted by Yuling Xing et al. identified a significant independent association between the TyG index and diabetes risk, particularly in females and individuals <65 years (18). While our findings similarly highlight the stronger association in participants <60 years, we observed no significant sex-specific interaction. This discrepancy may stem from our larger cohort (N = 155,337 vs. 5,575) and balanced sex distribution (74,622 males and 80,715 females). The mechanisms underlying the age - related heterogeneity in the TyG index effect remain unclear. A few studies have put forward that this phenomenon could be ascribed to the rapid societal changes. Presently, across China and the globe, the aging populace is on a continuous rise. Meanwhile, the long-standing family planning measures have led to a reduction in the labor force (35). This, in turn, has worsened the social burdens on the individuals younger than 60 years (36, 37).

While multiple mechanisms have been proposed to explain the TyG index predictive value for T2DM, the underlying mechanisms remain unclear. Pancreatic beta-cell dysfunction may contribute to the association between the TyG index and diabetes (38, 39). Beta cells are sensitive to glucotoxicity and lipotoxicity. Elevated glucose levels generate reactive oxygen species, which can cause cellular damage (40). Additionally, high blood triglyceride levels can increase ceramide and nitric oxide, suppressing muscle insulin activity and disrupting glucose uptake, ultimately leading to beta-cell death and insulin resistance (19, 41). These mechanisms may elucidate the correlation between the TyG index and diabetes risk. Several research suggest that aging promotes T2DM by impairing β-cell function and adaptability, resulting in compromised insulin secretion (42, 43). Certainly, further research is necessary to clarify these interactions.

This research presents several advantages, including the utilization of a comprehensive and large-scale database, the adjustment for numerous confounding factors, the observation of a threshold nonlinear association between the TyG index and diabetes, and the determination of the inflection point. Furthermore, we are the first to evaluate the increasing risk of newly developed diabetes associated with an elevated TyG index in healthy individuals with normal FPG and TG levels. However, this study also has several limitations, which are outlined as follows: (1) We did not distinguish between type 1 and type 2 diabetes, although our findings may be more relevant for assessing the risk of type 2 diabetes, given that approximately 95% of all diabetes cases are type 2 diabetes (44). (2) The database used in this study is fixed, which means that certain confounding variables, such as exercise status, may not have been included in our analysis. (3) Despite the data being collected by trained staff, some measurement errors may be inevitable, potentially affecting our analytical results. (4) The diagnosis of diabetes in this study was based on self-reported diagnoses and/or FPG levels ≥ 7 mmol/L. National surveys have indicated that isolated increased 2-hour plasma glucose following an oral glucose tolerance test (OGTT) accounts for 46.6% of undiagnosed diabetes cases in China. Consequently, the true prevalence of diabetes may be underestimated. (5) As our data were derived from a public database with specific exclusion criteria were predefined by its curators, we regrettably lack access to information regarding the excluded cohort. Strictly speaking, this methodological constraint may theoretically introduce selection bias.

## 5 Conclusion

In this study, we demonstrate that the TyG index has a significant independent association with diabetes in the general population, particularly among individuals with normal FPG and TG levels. This relationship is notably stronger in adults younger than 60 years. Our findings suggest that the risk of diabetes could be reduced by maintaining the TyG index below 8.41 through the management of FPG and TG levels. Overall, the TyG index may serve as a practical, affordable, reliable, and clinically applicable marker for diabetes. Given the rising incidence of diabetes and prevalence of undiagnosed cases, the TyG index could provide guidance for the general population to prevent the disease.

## Data Availability

The original contributions presented in the study are included in the article/supplementary material. Further inquiries can be directed to the corresponding authors.
